# Direct and residual antimicrobial effect of 2% chlorhexidine gel, double antibiotic paste and chitosan- chlorhexidine nanoparticles as intracanal medicaments against *Enterococcus faecalis* and *Candida albicans* in primary molars: an in-vitro study

**DOI:** 10.1186/s12903-023-02862-x

**Published:** 2023-08-04

**Authors:** Mariem Wassel, Mohamed Radwan, Reham Elghazawy

**Affiliations:** Pediatric Dentistry and Dental Public Health Department, Faculty of Dentistry, Ain Shams University Cairo, Organization of African Unity St.-Abbasia-Cairo, 11566 Cairo, Egypt

**Keywords:** Chitosan nanoparticles, Chlorhexidine, Double antibiotic paste, Intracanal medicaments, Primary molars, *Enterococcus faecalis*, *Candida albicans*

## Abstract

**Background:**

Thorough disinfection of root canals in primary molars may be complicated by the complex root canal morphology. This in-vitro study aimed to compare direct and residual antimicrobial effect of 2% chlorhexidine (CHX) gel, 500 mg/ml double antibiotic paste (DAP) and chitosan-chlorhexidine nanoparticles (CS-CHX NPs) as intracanal medicaments against *Enterococcus faecalis (E. faecalis)* and *Candida albicans (C. albicans)* in primary molars.

**Methods:**

Mesial roots of 63 mandibular second primary molars were infected with *E. faecalis and C. albicans*. Teeth were divided into 9 groups: Ia: (CS-CHX NPs), IIa: (CHX), IIIa: (DAP), IVa: chitosan nanoparticles (CSNPs) in which medicaments were placed for 3 days, groups Ib: (CS-CHX NPs), IIb: CHX, IIIb: (DAP), IVb: (CSNPs) in which medicaments were placed for 7 days, and Group V (control): teeth were infected, irrigated with saline, and sampled 3- and 7-days post-infection. Microbiological samples were obtained after infection, 3, and 7 days after medicament placement and 7 days after medicament removal for both time points. One-way ANOVA, Tukey’s post hoc test and paired t-test were used at p < 0.05.

**Results:**

CS-CHX NPs had the highest anticandidal effect which was comparable to CHX and significantly higher than other medicaments (p < 0.001) at both time points. CS-CHX NPs had the highest effect against *E. faecalis* which was comparable to DAP and significantly higher than other medicaments (p < 0.001) at 3 days. All medicaments showed similar effect against *E. faecalis* after 7 days. The 7-days placement significantly increased the antimicrobial effect against both micro-organisms in all groups, except CS-CHX NPs which showed an insignificant increase. CS-CHX NPs showed the highest residual effect against both micro-organisms that increased with 7-days placement.

**Conclusion:**

CSNPs and CHX combination showed a synergistic effect against both micro-organisms. CS-CHX NPs displayed a higher effect at a shorter period compared to other medicaments, yet its residual effect was higher with 7-days placement.

## Introduction

Pulpectomy is the treatment of choice for primary teeth with irreversible pulpitis and pulp necrosis [1]. The aim of pulpectomy is to eradicate infection and prevent its recurrence [2]. Root canal infections are polymicrobial in nature with domination of obligate and facultative Gram-positive anaerobes [2]. *Enterococcus faecalis* (*E. faecalis*) is a facultative anaerobic Gram-positive cocci which is frequently isolated from primary infected root canals, secondary infections or persistent endodontic infections [2, 3]. Its ability to adhere to and penetrate dentin as well as its biofilm forming ability, and tolerance to high pH values promote its capacity to resist a wide range of antimicrobials [4]. On the other hand, *Candida albicans* (*C. albicans*) is the prime fungus species that is commonly isolated from infected root canals especially in persistent root canal infections [5]. Its eradication may require a combination of antimicrobial irrigants, and intracanal medicaments [6, 7]. Like *E. faecalis*, virulence factors of *C. albicans* arise from its ability to infect dentin, invade dentinal tubules, and form resistant biofilms. It can also use dentin as a source of nutrition due its collagenolytic activity [6].

Bacterial-fungal interactions increase biofilm complexity and resistance to antimicrobials compared to planktonic cells. For example, *C. albicans* produces glucans that stabilize the 3D dimensions of biofilm while super-oxides produced by *E. faecalis* mediate hyphae production by *C. albicans* [8]. Furthermore, micro-organisms inside dentinal tubules and those in a biofilm are inaccessible to chemo-mechanical instrumentation. The outer layer of a microbial biofilm is usually removed by instrumentation and irrigation but micro-organisms in the innermost layers are protected by the biofilm extracellular matrix [2]. In primary molars, curved roots and anatomical complexity of root canal system can further undermine the efficiency of mechanical instrumentation rendering disinfection mostly relying on chemotherapeutics [9].

Root canal infections can be eradicated by a variety of antimicrobial irrigants and intracanal medicaments alongside mechanical instrumentation. However, it has been reported that medicaments have more potential to act inside infected dentinal tubules, lateral canals, and areas of root resorption [10, 11]. One of the most widely used intracanal medicaments is triple antibiotic paste (TAP) consisting of metronidazole, ciprofloxacin and minocycline. To avoid discoloration caused by minocycline, a double antibiotic paste (DAP) made of only metronidazole, and ciprofloxacin has been proposed which showed equal effectiveness as that of TAP [12–14] and a longer residual antibacterial effect [15]. Despite the effectiveness of antibiotic pastes for root canal disinfection, they lack an antifungal action which may necessitate the alternate use of an antifungal agent in combination with an antibiotic paste [16]. Another common root canal disinfectant, both as an irrigant or intracanal medicament, is chlorhexidine gluconate (CHX). It is a broad-spectrum cationic agent that possesses both antibacterial and antifungal properties and is characterized by high substantivity, and low toxicity compared to sodium hypochlorite. It is especially recommended in cases of open apex and root resorption. However, CHX is characterized by a low ability to dissolve organic substances or disrupt biofilms [17].

In recent years, chitosan which is a natural non-toxic polycationic polymer that is derived from chitin has gained increased interest in dentistry owing to its wide range of biological activities. It is distinguished for its biocompatibility, broad antibacterial and antifungal properties, adhesive properties, and biodegradability. It can also act as a drug delivery vehicle that enhances the therapeutic action of the loaded drug. Chitosan’s biodegradability provides a slow and controlled drug release that increases the loaded drug’s bioavailability while decreasing its toxicity [18]. Studies indicated that chitosan was effective against *E. faecalis* and *C. albicans* [19–21]. Interestingly, combining chitosan with other synthetic or natural products showed synergistic outcomes [18]. This was true when CHX was combined with chitosan of regular size in one study [22]. Furthermore, nanoparticles of chitosan were shown to possess a greater antimicrobial activity than parent chitosan due to the spherical shape of nanoparticles and greater surface area [23]. Yet, up to our knowledge, no studies evaluated the combination of chitosan nanoparticles (CSNPs) and CHX as an intracanal medicament.

Therefore, the aim of the current study was to compare the direct and residual antimicrobial effect of 2% CHX gel, DAP, and chitosan-chlorhexidine nanoparticles (CS-CHX NPs) gel as intracanal medicaments against *E. faecalis* and *C. albicans* in extracted primary molars.

## Materials and methods

A power analysis was designed based on a null hypothesis that there is no difference among tested groups. By adopting an alpha and beta levels of (0.05) i.e. power = 95% and an effect size (f) of (0.789) which were calculated based on the results of previous studies [24, 25]; the predicted sample size (n) was 63. (i.e. 7 samples per group).

### Preparation of intracanal medicaments

A clinically injectable DAP paste was prepared by mixing a 1:1 ratio of 500 mg ciprofloxacin (Ciprobay, Hikma pharma) and 500 mg metronidazole tablets (Flagyl, Sanofi aventis) with sterile distilled water to a concentration of 500 mg/ml [26, 27].

Polyvinylpyrrolidone (PVP), (Loba CHIME, India) was sprinkled gradually over 2% chlorohexidine digluconate solution (Gluco-CHeX; Cerkamed, Poland) under mild temperature with vigorous stirring to get a homogenous CHX gel.

**Fig. 1:**
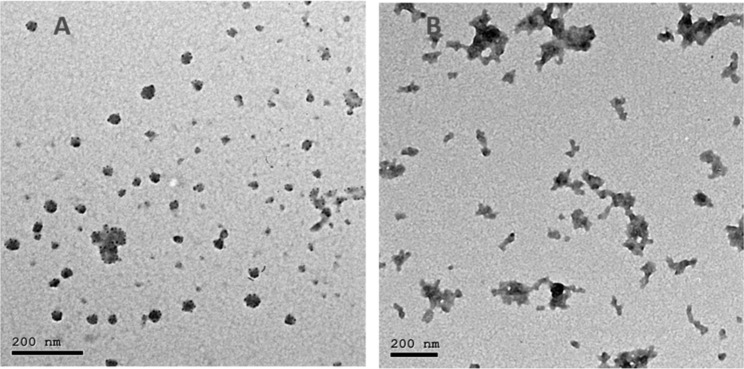
Transmission electron microscopy photos of (A): CSNPs, (B): CS-CHX NPs.

Chitosan nanoparticles (CSNPs) were prepared using the ionotropic gelation process [28]. Blank nanoparticles were obtained by the addition of a tripolyphosphate (TPP) aqueous solution (Merck Millipore, Germany) to a low molecular weight (˂100 kilodalton, DD = 85%) Chitosan (Loba Chemie, India) solution. One gram of chitosan powder was dissolved in 200 ml of 1% acetic acid (pH = 4) and stirred for 6-hours to get a homogenous solution. One hundred and fifty ml of 0.2% w/v TPP were added dropwise. When the clear solution turned to turbid indicating formation of CSNPs, the suspension was washed three times with DH_2_O by centrifugation for 30 min at 9000 rpm. To prepare the CS-CHX NPs, the prepared CSNPs were redispersed in 2% CHX solution with continuous stirring to get a chitosan concentration of 10 mg/ml. The produced nanoparticles had an average size of ˂ 50 nm and were spherical in shape as assessed by high resolution transmission electron microscopy (JEOL JEM-2100) at an accelerating voltage of 200 kV, Fig. 1. Thereafter, CS-CHX NPs and CSNPs gels were prepared using PVP as previously described.

The zeta potential of CSNPs and CS-CHX NPs was measured in triplite at 25 °C using Malvern Zetasizer (UK), version 8.02 after a 10X serial dilution in distilled water. The average zeta potential values for CSNPs and CS-CHX NPs were 23.66 mV and 13.7 mV, respectively.

### Teeth preparation

This in-vitro research was approved by the institutional research ethics committee (FDASU-Rec ER102205). Sixty-three mandibular second primary molars with no or minimal root resorption and patent mesio-lingual and mesio-buccal root canals were selected. Selected teeth had a root length of 10.2 ± 1 mm mesio-buccally and 9 ± 1 mm mesio-lingually. Teeth with coronal caries extending to the roots, severely destructed coronal structures that will not allow a temporary filling to be retained, or teeth with previous pulp therapy were excluded. Access cavities were made by high-speed round burs creating a direct access to the root canals. Coronal pulp tissue was removed by sterile excavators and each root canal patency was checked by # 15 K file (MANI INC, Japan). Mesial root canals were instrumented 1 mm shorter than the apical foramina using K files till file # 30 to standardize root canals’ diameter. Each root canal was irrigated with 10 ml of 5.25% NaOCL for 5 min followed by 10 ml of 17% EDTA solution (ENDO-SOLUTION, Cerkamed, Poland) for 5 min to remove organic and inorganic debris, respectively [29]. The canals were finally flushed with 20 ml saline for 5 min [29].

Teeth were autoclaved for two cycles at 121 °C for 15 min [24]. Each tooth was then incubated at 37 °C for 7 days in a sterile test tube with 5 ml sterile tryptone soy broth (TSB) (Oxoid LTD, England), and inspected daily for turbidity which, if present, indicates non-sterility [13]. To confirm sterility, all root canals were sampled and cultured on bile esculin agar (Acumedia/ Lab M, NEOGEN Culture Media, UK) and Sabouraud Dextrose agar (Oxoid LTD, England) for 24 h at 37 °C to detect any *E. faecalis* or *C. albicans* growth, respectively [13]. A water-resistant nail varnish was used to paint cementum and floor of pulp chamber. Cyanoacrylate was used to seal apical foramina of all roots and orifices of distal root canals.

### Root canals infection

*E. faecalis* (ATCC4083) was anaerobically grown on bile esculin agar for 24 h at 37 °C followed by suspending *E. faecalis* subcultures in brain heart infusion broth overnight. *C. albicans* (ATCC10231) was cultured on Sabouraud Dextrose agar for 24 h followed by suspension in TSB for 24 h at 37 °C. To prepare the root canals’ infecting solution, equal volumes of a one McFarland standard suspension prepared from each microbial suspension were mixed in a single sterile tube to produce a 1.5 × 10^8^ CFU/ml bacterial concentration corresponding to 0.5 McFarland standard [8, 29]. Mesial root canals were dried by sterile paper points. Subsequently, each canal was injected with 1 ml of the infecting solution. Each tooth was placed in a sterile test tube containing TSB and 100 µl of the infecting solution and incubated at 37 °C for 21 days during which each root canal was reinjected with 1 ml of the infecting solution on the fourth day of incubation [30]. After 3 weeks, each root canal was irrigated with 5 ml saline to remove bacterial suspension and teeth were sampled to assess baseline bacterial count (T0).

A study independent operator assigned teeth randomly into 9 groups (n = 7) according to treatment and duration of medicament placement as follows:

Group Ia: CS-CHX NPs (3days), group Ib: CS-CHX NPs (7days).

Group IIa: CHX (3 days), group IIb: CHX (7days).

Group IIIa: DAP (3 days), group IIIb: DAP (7days).

Group IVa: CSNPs (3 days), group IVb: CSNPs (7days).

Group V: Control group in which teeth were infected, irrigated with saline, and sampled 3- and 7-days post-infection.

Root canals were dried with sterile paper points and the intracanal medicaments were introduced using sterile 5 ml plastic syringes with disposable plastic tips until complete filling of the canals. Access cavities were sealed with sterile cotton pellets and a non-eugenol based temporary filling (Coltosol; Coltene-Whaledent, Cuyahoga Falls, OH, USA). Each tooth was wrapped in sterile gauze pads moistened with saline and incubated at 37 °C inside sterile test tubes. In the control group, teeth were immersed in 5 ml sterile TSB that was replenished every 3 days.

After 3 and 7 days of medicaments’ placement, the following samples were obtained by a blinded operator:

#### T3

Samples were obtained after 3 days of medicaments placement from groups Ia, IIa, IIIa, IVa and from control group.

#### T7

Samples were obtained after 7days of medicaments placement from groups Ib, IIb, IIIb, IVb and from control group.

After each medication period, a third sample was taken as follows:

#### T3’

Samples were obtained from groups Ia, IIa, IIIa, IVa seven days after medicaments removal.

#### T7’

Samples were obtained from groups Ib, IIb, IIIb, IVb seven days after medicaments removal.

Root canals were filled with sterile saline between T3 and T3’, and between T7 and T7’ [30].

At each sampling point, each root canal was irrigated with 20 ml saline, and two sequentially placed sterile #20 paper points were inserted in each root canal for 30 s [29]. The paper points of each tooth were placed in a single Eppendorf tube containing 1 ml saline and vortexed for 30 s. Saline was used as undiluted and 1/100 and 1/1000 dilutions. 10 µl of each dilution was plated on bile esculin agar and Sabaroud dextrose agar for 48 h at 37 °C for detection and quantification (CFU/ml) of *E. faecalis* and *C. albicans*, respectively. Grown colonies were identified by their morphology and confirmed by Gram staining. Data were analyzed using one-way ANOVA followed by Tukey’s post hoc test for intergroup comparisons and paired t-test for intragroup comparisons at p < 0.05.

## Results

Results for percent (%) reduction in microbial count after 3 and 7 days of medicament placement are presented in Table (1). For *C. albicans*, there was a significant difference between values of different groups at both time intervals. Post hoc pairwise comparisons showed CS-CHX NPs and CHX to have significantly higher values than other groups (p < 0.001). CSNPs showed a significantly higher percent reduction (p < 0.001) compared to DAP. Whereas the control group had the lowest percent reduction value.

**Table 1:** Microbial count % reduction after 3 and 7 days of medicaments placement

Micro-organism	Interval	Microbial count reduction (%) (Mean ± SD)					f-value	p-value
		***CS-CHX NPs***	***CHX***	***DAP***	***CSNPs***	***Control group***		
*C. albicans*	***(T3-T0)***	99.34 ± 0.85^ A^	93.06 ± 2.09^ A^	8.27 ± 3.97^ C^	53.80 ± 11.85^B^	-5.01 ± 5.76^D^	**269.30**	< 0.001*
	***(T7-T0)***	99.97 ± 0.01^ A^	98.49 ± 0.43^ A^	23.07 ± 5.47^ C^	69.93 ± 8.21^B^	-10.87 ± 7.87^D^	**352.51**	< 0.001*
	***t-value***	**1.66**	**6.34**	**4.44**	**4.06**	**1.37**		
	***p-value***	**0.173**	0.003*	0.011*	0.015*	**0.264**		
*E. faecalis*	***(T3-T0)***	99.96 ± 0.03^ A^	95.74 ± 0.81^B^	99.68 ± 0.06^ A^	86.61 ± 1.96^ C^	0.00 ± 0.04^D^	**8204.85**	< 0.001*
	***(T7-T0)***	99.96 ± 0.05^ A^	99.04 ± 1.17^ A^	99.96 ± 0.07^ A^	93.39 ± 3.52^ A^	-5.47 ± 7.89^B^	**705.10**	< 0.001*
	***t-value***	**0.46**	**6.34**	**15.21**	**4.27**	**1.38**		
	***p-value***	**0.671**	0.003*	< 0.001*	0.013*	**0.262**		

Means with different superscript letters within the same horizontal row are significantly different *significant (p < 0.05).

For CHX, DAP and CSNPs groups, the reduction measured at  (T7-T0) was significantly higher than that measured at (T3-T0) (p < 0.05). For other groups, the difference was not statistically significant (p > 0.05).

For *E. faecalis*, post hoc pairwise comparisons for (T3-T0) showed CS-CHX NPs and DAP to have significantly higher values than other groups (p < 0.001). In addition, analysis showed CHX, CSNPs and control group to be significantly different from each other (p < 0.001) with CHX having the highest value followed by CSNPs, while the control group had the lowest value. For (T7-T0), post hoc pairwise comparisons showed insignificant differences among all groups except for control group which had significantly lower value than other groups (p < 0.001).

For CHX, DAP and CSNPs groups, the reduction measured at (T7-T0) was significantly higher than that measured at (T3-T0) (p < 0.05). For other groups, the difference was not statistically significant (p > 0.05).

Results for percent (%) change in microbial count one week after medicaments removal are presented in Table (2). For *C. albicans*, there was a significant difference among different groups at (T3’-T3) and (T7’-T7) (p < 0.001). Post hoc pairwise comparisons for (T3’ -T3) showed CS-CHX NPs to have significantly higher values than CSNPs, CHX, and DAP groups (p < 0.001). Additionally, CSNPs and CHX showed significantly higher values than DAP (p < 0.001). For (T7’-T7), post hoc pairwise comparisons showed CS-CHX NPs to have significantly higher value than other groups (p < 0.001). Also, CSNPs was found to have significantly higher value than CHX and DAP (p < 0.001). For CSNPs, the reduction measured at(T7’-T7) was significantly higher than that measured at (T3’-T3) (p < 0.001). For other groups, the difference was not statistically significant (p > 0.05).

**Table 2:** Microbial count % change one week after medicaments removal

Micro-organism	Interval	Microbial count reduction (%) (Mean ± SD)				f-value	p-value
		***CS-CHX NPs***	***CHX***	***DAP***	***CSNPs***		
*C. albicans*	***(T3’-T3)***	87.00 ± 12.04^ A^	17.70 ± 14.88^BC^	6.17 ± 4.30^ C^	25.00 ± 0.00^B^	**68.09**	< 0.001*
	***(T7’-T7)***	93.50 ± 6.02^ A^	5.35 ± 3.66^ C^	2.96 ± 2.87^ C^	63.57 ± 2.72^B^	**610.77**	< 0.001*
	***t-value***	**1.12**	**1.64**	**1.13**	**31.69**		
	***p-value***	**0.324**	**0.177**	**0.321**	< 0.001*		
*E. faecalis*	***(T3’-T3)***	91.25 ± 0.88^ A^	10.26 ± 0.10^BC^	5.94 ± 5.95^ C^	25.53 ± 5.95^B^	**69.12**	< 0.001*
	***(T7’-T7)***	91.90 ± 5.18^ A^	10.34 ± 8.93^B^	11.07 ± 2.78^B^	86.08 ± 4.08^ A^	**387.37**	< 0.001*
	***t-value***	**0.29**	**0.02**	**2.19**	**6.87**		
	***p-value***	**0.784**	**0.986**	**0.093**	0.002*		

Means with different superscript letters within the same horizontal row are significantly different *significant (p < 0.05).

Regarding *E. faecalis*, significant difference was found among different groups at (T3’-T3) and (T7’-T7) (p < 0.001). Post hoc pairwise comparisons for (T3’-T3) showed CS-CHX NPs to have significantly higher values than other groups (p < 0.001). CSNPs was also found to have significantly higher value than DAP (p < 0.001) but not CHX. For (T7’-T7), post hoc pairwise comparisons showed CS-CHX NPs and CSNPs groups to yield significantly higher values than other groups (p < 0.001), while CHX and DAP showed comparable effects. For CSNPs, the reduction measured at (T7’-T7) was significantly higher than that measured at (T3’-T3) (p = 0.002). For other groups, the difference was not statistically significant (p > 0.05).

## Discussion

Single rooted teeth were commonly utilized in in-vitro studies assessing intracanal medicaments in primary teeth. In the current study, mesial roots of primary molars were used to assess the action of the experimental medicaments in primary molars in which accessory canals and horizontal anastomoses are frequently located [9]. It was reported that second primary molars’ accessory canals are mainly found in the root surface of furcation area while those of the first primary molars are mainly located within the area of root division [31].

In the present study, microbiological samples were obtained after 3 and 7 days of medicaments’ placement using paper points to give a chance for micro-organisms inside dentinal tubules and accessory canals, if present, to proliferate and refill canals’ lumens so as to assess the residual effect of the medicaments after their removal [30, 32].

Results of the present investigation indicated that CS-CHX NPs and CHX were the most effective against *C. albicans* at both time points. For *E. faecalis*, CS-CHX NPs showed the highest effect after 3 days which was equivalent to DAP and significantly higher than CHX and CSNPs. Yet, all medicaments exhibited a comparable effect against *E. faecalis* after 7 days with CS-CHX NPs and DAP still showing the highest effect.

The effectiveness of 2% CHX as a root canal medicament has been confirmed by many studies [17, 25, 33, 34]. Nevertheless, in these studies 2% CHX was not able to completely eradicate *E. faecalis*. Inability of CHX to completely eradicate *C. albicans* was as well reported [35]. It was also reported that CHX was significantly more effective against *E. faecalis* than calcium hydroxide [36] which may be related to *E. faecalis’* ability to tolerate high pH (9–11) by virtue of their proton pump that can acidify the cytoplasm even in an alkaline pH [12].

Kumar et al. [34] revealed that 2% CHX was the most effective against *E. faecalis* inside dentinal tubules at 200 and 400 μm depths compared to calcium hydroxide and bamboo salt. Similar to the present study, the authors reported that placement of 2% CHX for 7 days increased its action against *E. faecalis* specially at 400 μm depth. The authors also reported that 2% CHX was able to penetrate up to 861 μm into dentin, yet the reported depth of penetration of *E. faecalis* in the same study was 821.91 to 1061.79 μm.

Clinical studies also supported the effectiveness of CHX as a root canal medicament. One study reported that placing 2% CHX for 7 days in single rooted permanent teeth had the highest percent reduction of facultative anaerobes, obligate anaerobes and *Candida* species compared to calcium hydroxide [37]. Whereas a clinical study that compared the effect of 1% CHX, calcium hydroxide or their combination as root canal medicaments in single rooted primary teeth, reported that all medicaments significantly reduced *E. faecalis* after 48 h [38]. On the contrary, a clinical study by Paikkatt et al. [11] revealed that 1% CHX gel, calcium hydroxide, and 1% metronidazole placed for two weeks were ineffective as root canal medicaments in significantly reducing counts of aerobic and facultative anaerobic micro-organisms in single rooted necrotic primary teeth.

The antimicrobial action of CHX is related to its positive charge which gives CHX a wide spectrum of antimicrobial action. CHX’s positive charge interacts with the negatively charged phosphate group in microbial cell membrane increasing cell membrane permeability and causing leakage of low molecular weight ions as phosphorus and potassium ions. At high concentrations, precipitation of cytoplasmic contents leads to cell death [17]. Moreover, the unique substantivity of CHX allows for a prolonged antimicrobial activity [39].

The potent antimicrobial effect of CSNPs perse was previously reported [8, 23, 40, 41]. CSNPs possess greater antimicrobial action than chitosan and chitin due to their spherical particle shape that leads to a smaller particle size, high surface area and greater reactivity [23, 42]. The higher reactivity of the polycationic nanoparticles allows a greater degree of interaction with the negatively charged microbial cell wall. Additionally, the larger surface area allows a tighter adhesion to microbial cell wall. Subsequently, disruption of cell wall, leakage of intracellular contents and cell death ensue [23]. Other probable antimicrobial mechanism of CSNPs includes metal chelation that reduces the availability of essential nutrients and hence reduces cell growth. Moreover, CSNPs can penetrate microbial cell wall due to their small particle size and subsequently bind to microbial DNA preventing its transcription [23, 40].

A study by Kishen et al. [41], verified that CSNPs with a 70 nm diameter and a zeta potential of 49 mV caused complete eradication of *E. faecalis* after 8 h when assessed by the direct-contact assay against planktonic cells. In the same study, zinc-oxide nanoparticles and a nanoparticulate combination of chitosan and zinc-oxide showed a 3- 4-fold log reduction in bacterial count. Another study by Sireesha et al. [43] revealed that CSNPs were more effective against *E. faecalis* compared to chitosan using the agar diffusion test. Moreover, CSNPs were able to diffuse deeper into dentinal tubules compared to chitosan. The authors reported that the penetration depth of CSNPs into dentinal tubules was 477.02 μm. It is worth to mention that the penetration depth of CSNPs (477.02 μm) in the study of Sireesha et al. [43] was lower than that reported for CHX (861 μm ) in the study of Kumar et al. [34]. Sireesha et al. [43] stated that the low penetration depth of CSNPs was related to the gelling-out tendency of CSNPs which toughened its handling. Although in the present study penetration depth of medicaments was not assessed, yet, gelling out of CSNPs or CS-CHX NPs did not occur which may be related to the differences in the manufacturing process or the study design. For an instance, Sireesha et al. [43] did not report the molecular weight of the used chitosan to prepare the CSNPs, which has a direct correlation with the viscosity of the final product, nor the duration of medicaments placement.

In the present study, a synergistic effect was found in CS-CHX NPs group where the antimicrobial action of CS-CHX NPs against both micro-organisms was higher than each agent alone. In agreement with our results, CSNPs showed a synergistic antimicrobial effect when loaded with ozonated olive oil [8], propolis [44], calcium hydroxide [45], and ciprofloxacin [46], supporting its effectiveness as a drug carrier. Conversely, the antimicrobial action of CHX was reduced when it was combined with calcium hydroxide and other medicaments [36, 47]. Nevertheless, a clinical study demonstrated a synergistic effect against *E. faecalis* when a combination of CHX and chitosan of regular size was used as an intracanal medicament for 7 days in single rooted permanent teeth with failed endodontic treatment. The authors reported that complete elimination of *E. faecalis* was not achieved, yet an 83% reduction in *E. faecalis* count was evident in CHX-chitosan group compared to 73% for 2% CHX gel and 71% for 2% chitosan gel [22]. Another in-vitro study demonstrated that CHX and chitosan combination generated the highest inhibition zones for *E. faecalis* and *C. albicans* compared to 2% chitosan gel and 2% CHX gel [48].

One drawback of CHX is its inability to disrupt biofilms [17]. Thus, the noted synergistic effect may be related to the antibiofilm potential of CSNPs where it has been found that CSNPs can inhibit biofilm formation by inhibiting extracellular polysaccharide synthesis [23, 46]. At the same time, CSNPs were also found to reduce formed biofilms’ mass due to their increased penetrability into biofilm channels [41, 46, 49]. Additionally, the small particle size of CS-CHX NPs is another important influencing factor. It has been reported that a particle size < 500 nm is needed to access dentinal tubules [50] and disrupt dental biofilm [46]. Parolia et al. [44] showed that chitosan-propolis nanoparticles were effective at reducing *E. faecalis* count inside dentinal tubules at 200 and 400 μm depths. The authors stated that the small particle size of the prepared combination (107.74 ± 0.53 nm) allowed penetration into dentinal tubules.

One more influencing factor on the antimicrobial effectiveness of nanoparticles is the zeta potential of the formed particles. Zeta potential measures the surface charge of the formed nanoparticles and indicates the long-term stability of dispersion of colloidal systems [51]. To prevent self-aggregation of nanoparticles, a zeta potential of ± 30 mV is needed so that a strong electrostatic repulsion is created among nanoparticles [52]. Yet, a positive zeta potential promotes drug delivery to the negative microbial cell membrane through enhancing drug adherence [49]. In the present study, the zeta potential of CSNPs and CS-CHX NPs was lower than + 30 mV which may be related to the addition of PVP which is negatively charged. Reduced zeta potential in our study could also be related to using low molecular weight chitosan to produce the nanoparticles or to the low concentration of the final formulations [40]. Adding CHX to CSNPs further reduced the zeta potential of CHX-CS NPs.  This lower net positive charge of CHX-CS NPs may be related to the addition of CHX that masked some areas of the polycationic CSNPs after conjugation. Therefore, the noted antimicrobial effect of CSNPs and CS-CHX NPs in the current study could be largely related to their small particle size. This assumption can be supported by the study of Ing et al. [40] who concluded that both the zeta potential and particle size of CSNPs were directly related to the molecular weight of the parent chitosan as well as the concentration of CSNPs solution. The authors also stated that CSNPs prepared from low molecular weight chitosan had the lowest particle size and zeta potential, yet the highest antifungal effect.

Using CSNPs as a carrier system also could have allowed a controlled sustained release of the loaded CHX and thus ensured a prolonged activity of CHX. Ballal et al. [48] confirmed that chitosan enhanced the release of CHX over a prolonged time period by testing CHX release from a CHX-chitosan combination using UV spectrophotometer. The authors reported that the rate of CHX release from this combination was better than that from a 2% CHX gel. Another study reported that ciprofloxacin release from ciprofloxacin-Poly (lactic-co-glycolic acid) nanoparticles coated with chitosan was 78.3% over 72 h, while a 100% release was evident from a ciprofloxacin solution at the first hour [46]. Thus, in the present study, the controlled release property of CSNPs, and substantivity of CHX may have maintained the antimicrobial action for longer periods compared to other medicaments. An important advantage of a prolonged antimicrobial action is reduction of the risk for developing resistant microbial strains [22].

DAP in the current study showed the weakest antifungal effect which was consistent with other studies [16, 53]. To increase the antifungal effect of antibiotic pastes, various additives were used such as chitosan [16] and antifungal drugs [53]. Contrarywise, DAP efficiency against *E. faecalis* was high agreeing with various studies [12–14, 54, 55]. Results also showed that DAP effect against *E. faecalis* was comparable to CSNPs at both time intervals.

The 7-days placement significantly increased the effect against both micro-organisms in CHX, DAP and CSNPs groups. Interestingly, only CS-CHX NPs had a comparable antimicrobial effect at 3 and 7 days against both micro-organisms indicating a considerable potency in a shorter duration. This coincides with the results of Parolia et al. [44] who reported that after one and three days of chitosan-propolis nanoparticles application, a significantly higher reduction of *E. faecalis* count at 200 and 400 μm depths of dentinal tubules was evident compared to propolis, chitosan and 2% CHX gel. However, after 7 days, comparable results were evident in all groups specially at 400- µm depth. Notably, the residual effect of CS-CHX NPs against *C. albicans* in the present study was higher after a 7-days placement compared to 3-days. Furthermore, DAP placement for 7 days increased the residual effect against *E. faecalis*. From these findings, it can be deduced that a 7-days medicaments’ placement is more favorable for all medicaments.

The residual effect of an antimicrobial agent refers to the ability of this agent to bind to tooth structure and be released over a prolonged period in an active state. Residual effect of antimicrobials can inhibit reinfection of the root canals from micro-organisms inside dentinal tubules. Table 2 demonstrates that CS-CHX NPs followed by CSNPs showed the highest residual effect against both micro-organisms. This residual effect increased with 7-days medicament’s placement compared to 3-days placement. The possible enhanced penetrability of nanoparticles into dentinal tubules and accessory canals, the sustained release property of CSNPs, a probable increased adhesiveness of CSNPs to dentin, as well as the substantivity of CHX are all factors that may have contributed to a prolonged bioavailability of both agents, thus increasing their residual antimicrobial activity. This highlights the need for investigating such factors in future studies.

On the other hand, the 7-days placement of CHX and DAP increased the residual effect against *E. faecalis*, while it decreased that against *C. albicans*. Previous studies reported that both 2% CHX [30, 56] and DAP [15, 27] demonstrated an antimicrobial residual effect. Similar to our study, Valera et al. [30] revealed that 2% CHX demonstrated a higher residual effect against *E. faecalis* compared to that against *C. albicans* when used as an intracanal medicament for 14 days. In that study, total elimination of both micro-organisms was not achieved neither after medicament placement nor 7-days after medicament removal. CHX substantivity is related to its positive charge which gives CHX the ability to inhibit reinfection of dentinal tubules. An in-vitro study reported that 2% CHX was able to prevent reinfection of dentinal tubules for up to 21 days following its application for 7 days [56].

The positive charge of chitosan and CHX allows adherence to the negatively charged dentin, so that when micro-organisms attempt to reinfect the root canal, the positively charged agents interacts with the negatively charged microbial cell wall causing their destruction. This may explain the residual effect noted with CSNPS, CS-CHX NPs, and CHX. These findings were in line with Kishen et al. [41] who reported that dentin irrigated with CSNPs, chitosan-zinc oxide nanoparticles or CHX produced a significant reduction in *E. faecalis* adherence to dentin as assessed by fluorescence microscopy.

Jenks et al. [27] reported that the antibacterial effect of a 500 mg/ml DAP concentration applied for one week onto radicular dentin was efficient against 3weeks-old *E. faecalis* biofilm. Furthermore, this concentration caused a significantly higher residual antibiofilm activity against *E. faecalis* compared to the concentrations commonly used for regeneration (1, 5, 50 mg/ml). Since in our study we aimed to assess medicaments’ effectiveness for pulpectomy in primary teeth, a 500 mg/ml DAP concentration was used, specially that it was reported that when a short treatment time is used, high concentrations are preferred to obtain an extended residual effect [27]. Sabrah et al. [15] suggested that ciprofloxacin and metronidazole may have the ability to penetrate into dentin due to their low molecular weight. The authors also stated that DAP is harder to remove from root canals compared to TAP due to the higher solubility of minocycline compared to ciprofloxacin which therefore may increase DAP’s residual effect compared to TAP.

## Conclusion

- The combination of CSNPs and CHX showed a synergistic action and displayed the highest effect against *C. albicans* and *E. faecalis* at both time points.
- At both time points, CS-CHX NPs showed a comparable effect to CHX against *C. albicans* and a comparable effect against *E. faecalis* as that of DAP. However, CS-CHX NPs achieved a higher antimicrobial action in a shorter duration and the residual effect of CS-CHX NPs was higher than other antimicrobial agents.
- The 7-days medicaments’ placement increased the direct antimicrobial action of the tested agents and the residual effect of CS-CHX NPs and DAP.

## Data Availability

Datasets of the current study are available from the first author (Wassel M) upon request.
